# Retinal fluid changes and therapeutic effects in symptomatic circumscribed choroidal hemangioma patients: a long-term follow up study

**DOI:** 10.1186/s12886-018-0993-x

**Published:** 2018-12-13

**Authors:** Junwon Lee, Christopher Seungkyu Lee, Min Kim, Sung Chul Lee

**Affiliations:** 1 Department of Ophthalmology, Eye and ENT Hospital, Severance Hospital, Institute of Vision Research, Yonsei University College of Medicine, 50-1 Yonsei-ro, Seodaemun-gu, Seoul, 03722 South Korea; 2 Department of Ophthalmology, Institute of Human Barrier Research, Gangnam Severance Hospital, Yonsei University College of Medicine, Seoul, South Korea

**Keywords:** Choroidal hemangioma, Photodynamic therapy, Cystoid macular oedema, Retinal fluid pattern

## Abstract

**Background:**

Changes in retinal fluid patterns associated with circumscribed choroidal hemangioma (CCH) have not been investigated yet. A long-term follow-up study was performed to evaluate the changes of retinal fluid patterns and treatment responses.

**Methods:**

We retrospectively reviewed medical records of all CCH patients diagnosed between November 2005 and March 2017. Enrolled patients had visual symptoms, were treatment-naïve, and had been followed-up for more than 2 years. Best corrected visual acuities (BCVA) and the presence, severity, and pattern change of the subretinal fluid (SRF) and intraretinal fluid (IRF) in the macula on optical coherence tomography (OCT) were analyzed at initial presentation and follow-up visits.

**Results:**

Twenty-six patients were enrolled. All patients received one or more of the following treatments: PDT, TTT, and intravitreal bevacizumab (Avastin) injection (IVB). Primary therapy consisted of PDT in 9 patients (34.6%), TTT in 7 patients (26.9%) and IVB in 10 patients (38.5%). At initial presentation, the SRF-only pattern was mostly observed. Despite treatment, IRF occurred over time; eventually, advanced cystoid macular oedema (CME) developed. In terms of retinal fluid reduction, PDT was most efficacious (9/9, 100%), and TTT and IVB showed moderate efficacy (TTT: 4/7, 57.1%; IVB: 5/10, 50%) as a primary therapy. After advanced CME developed, IVB and TTT showed no or minimal effect (TTT: 0/1, 0%; IVB: 0/19, 0%), and PDT was the only effective therapy (6/10, 60%).

**Conclusion:**

The pattern of retinal fluid accompanied by CCH evolved from an SRF-only pattern initially to an advanced CME pattern. The effectiveness of treatments decreased over time, and advanced CME generally showed resistance to treatments. PDT would be the most recommended treatment.

## Background

Circumscribed choroidal hemangioma (CCH) is a benign tumour, and asymptomatic CCH does not require treatment. Associated serous retinal detachment and cystoid macular edema (CME) are common findings in symptomatic CCH. Various treatment modalities including photodynamic therapy (PDT) [1–7], transpupillary thermotherapy (TTT) [8–10], anti-vascular endothelial growth factor (VEGF) injections [8], lens-sparing external beam radiotherapy [11, 12], plaque brachytherapy [13, 14], proton beam therapy [15], stereotactic radiosurgery [16], laser photocoagulation [11], and oral propranolol [17] have been applied for treating CCH related symptomatic fluids. Although various treatments have been explored, according to recent studies, PDT has emerged as the treatment of choice with high rates of tumour regression, fluid resorption and minimal complications [4–7, 18].

In a large study, serous retinal detachment and CME were reported in 81 and 17% of patients at initial presentation, respectively [19]. However, there have been no longitudinal observational studies to investigate changes in retinal fluid patterns associated with CCH.

The aim of this study was to evaluate the changes in retinal fluid patterns, the response to various treatment modalities, and the prognosis of visual acuity in patients with CCH through a long-term follow-up.

## Methods

We retrospectively reviewed the medical records of all patients diagnosed with CCH at Yonsei University Severance Hospital between November 2005 and March 2017. Patients who had visual symptoms, were treatment-naïve at initial presentation, and were followed-up for more than 2 years were enrolled. CCH was diagnosed based on fundus examination, indocyanine green angiography (ICGA), and ultrasonography. This study was approved by the Institutional Review Board at Yonsei University Medical Center (Reference No. 4–2017-0955) and adhered to the tenets of the Declaration of Helsinki.

Baseline demographic data, including age, sex, general and ocular history, symptoms, follow-up duration, and series of treatments were recorded.

Initial assessment of patients included the minimal angle of resolution (logMAR) best-corrected visual acuity (BCVA) measurement using the Snellen visual acuity chart, slit-lamp biomicroscopy, dilated fundus examination, fundus photography, fluorescein angiography and ICGA, B-scan ultrasonography, and optical coherence tomography (OCT) (Stratus III OCT, Carl Zeiss, Dublin, California, USA; or Spectralis HRA + OCT, Heidelberg Engineering, Heidelberg, Germany).

Tumour size and location, and distance to the foveola and optic disc margin were measured using B-scan ultrasonography, fundus photography, and OCT.

BCVA, and the presence, severity, and pattern change of the retinal fluid on OCT were observed during every follow-up visit. Changes in retinal fluid following each treatment were evaluated qualitatively and quantitatively.

The pattern of retinal fluid in the macula was divided into three types; subretinal fluid (SRF) only; SRF / intraretinal fluid (IRF) combined; and advanced (well organized) CME pattern consisting of severe IRF.

### Treatment modalities

“All patients received one or more of the following treatments: PDT, TTT, and intravitreal bevacizumab (Avastin) injection (IVB).”

PDT was performed with standard equipment under standard conditions. An 83-s laser spot at 689 nm (50 J/cm^2^) coupled with intravenous verteporfin was used to treat the entire CCH. Verteporfin 6 (typical) or 12 mg/m^2^ (enhanced) was injected. The area of treatment was determined by the size of the tumour measured using ICGA. When the target area exceeded the maximum spot size, treatments were repeated several times without overlap.

TTT was performed under topical anesthesia via a dilated pupil. Patients were treated with an infrared diode laser at 810 nm using a slit-lamp biomicroscope delivery system. Each tumour was covered entirely with confluent laser spots, with the power ranging from 200 to 500 mW and spot size between 1200 and 3000 μm to induce a slight color change with 1 min of exposure at each spot. An area of 1-disc diameter (DD) around the foveola and 1 DD around the disc margin were spared during TTT in all cases.

Using an aseptic technique, bevacizumab (Avastin) 1.25 mg was injected 3.0 or 3.5 mm posterior to the limbus through the pars plana using a 30-gauge needle in the operating room.

### Comprehensive evaluation of therapeutic response

Indications for treatment included serous macular detachment (SMD) and CME causing visual symptoms. When the fluid was fully resolved, no further treatment was performed. When the treatment effect was insufficient or the fluid recurred, treatment was performed again. When the treatment appeared to be ineffective or an additional session using the same treatment modality had the potential to induce retinal damage, a different therapy was trialled. OCT was performed within 8 weeks after each treatment. The treatment effect was evaluated after 4 weeks in cases where IVB was administered. In cases where patients received PDT or TTT treatment, monitoring continued for at least 8 weeks.

During long-term follow-up, the types, order, and number of treatments administered varied among patients. For analysis, the series of treatments were simplified. The efficacy of each treatment was assessed by whether it increased or decreased the amount of SRF and/or IRF on OCT. Efficacy was classified into three groups and was numerically parameterized.

A reduction of less than 20%, or no change, in the fluid following treatment was classified as ‘minimal or none’ and parameterized as 0 point. For cases with a 20 to 80% reduction following treatment, these were classified as ‘partial’ responses and parameterized as 0.5 point. For cases with an 80 to 100% reduction, these were classified as ‘almost or complete’ responses and parameterized as 1 point. The mean score for several sessions of a single treatment modality was obtained when the same treatment modality was applied continuously in a series.

For example, if IVBs were performed 5 times consecutively and the effects were #1: almost or complete; #2: partial; #3: minimal or none; #4: minimal or none; and #5: partial, the overall mean efficacy of IVB was 0.4 (1.0 + 0.5 + 0 + 0 + 0.5 / 5 = 2.0 / 5).

### Statistics

Statistical analyses were performed using SPSS 23.0 software (IBM Corp., Armonk, NY, USA). Averages are reported as the mean ± standard deviation (SD). When comparing the paired mean at different points within an individual, a paired t-test was performed. A *p*-value of < 0.05 was considered statistically significant.

## Results

All enrolled patients had symptomatic CCH and were treatment-naïve. All patients received PDT and/or TTT and/or IVB for serous macular detachment and CME. Patient demographics and clinical data are shown in Table 1.

**Table 1:** Patient demographics, tumor characteristics, changes in retinal fluid, series of treatments and best corrected visual acuities of patients with circumscribed choroidal hemangioma

Case No.	Age (years)/ Sex	FU (months)	Foveal distance (mm)	Initial LBD (mm)	Initial height (mm)	Initial retinal fluid pattern	IRF occurrence (Y/N)	Organized IRF pattern (Y/N) / Time point after initial presentation (mo)	Series of treatments (type-No.)	Initial BCVA (Snellen)	Highest BCVA during FU (Snellen)	BCVA change, Last BCVA (Snellen)
1	45/M	129	0	9	3.7	SRF only	Y	Y / 86	T3-A10-P1	0.5	0.8	Worsen, 0.01
2	66/F	123	0	10.1	4.6	SRF only	Y	N	T1-A2-T	0.05	0.1	Worsen, HM
3	57/M	114	0	9.4	3.5	SRF only	Y	N	T3-A2	0.2	0.2	Worsen, CF
4	52/F	110	5	9.3	4.9	SRF + IRF	Y	N	T1	0.025	0.025	Worsen, 0.01
5	63/M	96	1.5	8.27	3.22	SRF + IRF	Y	Y / 72	T2-A3-P1(E)	0.16	0.4	Worsen, 0.025
6	44/F	90	4	9.17	3.74	SRF only	Y	Y / 39	A2-P2-P1(E)	0.63	1.0	Improved, 0.8
7	48/F	79	0.75	11.07	4.77	SRF only	Y	Y / 71	A2-T1-P1-P1(E)	0.63	0.8	Worsen, 0.04
8	47/F	71	0	9.5	3.73	SRF only	N	N	A2-P1	0.2	0.63	Improved, 0.63
9	39/M	70	0	10.6	2.96	SRF only	Y	Y / 11	A2-P2-A3-P1(E)	0.63	0.8	Worsen, 0.16
10	51/F	65	2.2	8.4	3.2	SRF + IRF	Y	Y / 23	T2-A4-T1-A2	0.01	0.01	Worsen, CF
11	50/F	60	3	10.43	3.83	SRF + IRF	Y	N	P2	0.16	0.8	Improved, 0.8
12	60/M	59	1.3	7.99	2.27	Advanced CME	Y	Y / 0 (initial)	A2-P2-A1	0.63	0.8	Worsen, 0.4
13	52/F	59	1.6	5.24	1.95	SRF only	Y	N	P1(E)	0.63	1.0	stable
14	55/M	57	1	6.84	2.01	SRF only	Y	N	A5-P1(E)	0.2	0.63	Improved, 0.32
15	35/F	57	0	10.5	5.21	Advanced CME	Y	Y / 0 (initial)	A1	0.025	0.08	stable
16	35/F	55	2.9	10.53	4.73	SRF only	N	N	T2	0.05	0.2	Worsen, 0.013
17	54/M	54	0	11.86	3.64	Advanced CME	Y	Y / 0 (initial)	A1-P1-P1(E)-A3	0.4	0.5	Worsen, 0.063
18	57/M	36	0	8.91	2.24	SRF only	N	N	P1	0.2	0.32	Improved, 0.32
19	23/M	36	0.75	6.75	2.00	SRF only	N	N	P1-A2	0.32	0.63	Worsen, 0.04
20	40/M	36	3.5	8.73	3.37	SRF only	Y	N	P1- P1(E)	0.05	0.8	Improved, 0.32
21	47/M	34	0	7.36	2.91	SRF only	N	N	A1-P1(E)	0.013	0.32	Improved, 0.1
22	62/M	33	0	4.56	1.52	SRF only	N	N	P1	0.2	0.32	Stable
23	40/M	31	0	7.81	2.36	SRF only	N	N	P1-A2-P1	0.32	0.5	stable
24	63/M	33	3.2	7.29	2.64	SRF only	Y	N	A2-P2(E)	0.25	0.8	Improved, 0.5
25	49/M	42	0	8.14	2.98	SRF only	N	N	P1(E)	0.2	0.2	Worsen, 0.16
26	46/M	28	1.2	8.52	3.97	SRF only	N	N	P1-P1(E)	0.4	0.8	Improved, 0.5

*No.* number, *FU* follow-up duration, *LBD* largest base diameter, *SRF* subretinal fluid, *IRF* intraretinal fluid, *CME* cystoid macula oedema

*BCVA* best corrected visual acuity, *M* male, *F* female, *HM* hand motion, *LP-* no light perception, *CF* counting finger

*T* transpupillary thermotherapy, *P* photodynamic therapy (typical dose), *A* intravitreal bevacizumab (Avastin) injection, *P(E)* enhanced dose P

Twenty-six patients were enrolled. The mean ± SD (minimum to maximum) follow-up duration was 63.68 ± 30.10 (range, 27.77 to 128.52) months. The median and interquartile ranges of follow-up duration were 58.33 months and 35.73 to 82.02 months, respectively. Seventeen patients were followed-up for 48 months and 9 patients were followed up for 24 to 48 months.

The mean age was 49.26 ± 10.07 years. There were 16 males and 10 females. The mean largest base diameter (LBD) and height of the tumour were 8.70 ± 1.73 (range, 4.56–11.86) and 3.31 ± 1.02 (range, 1.52–5.21) mm, respectively. Twelve tumours involved the subfoveal area and 14 tumours were located in the extrafoveal area.

### Pattern change of retinal fluid related with CCH

**Fig. 1:**
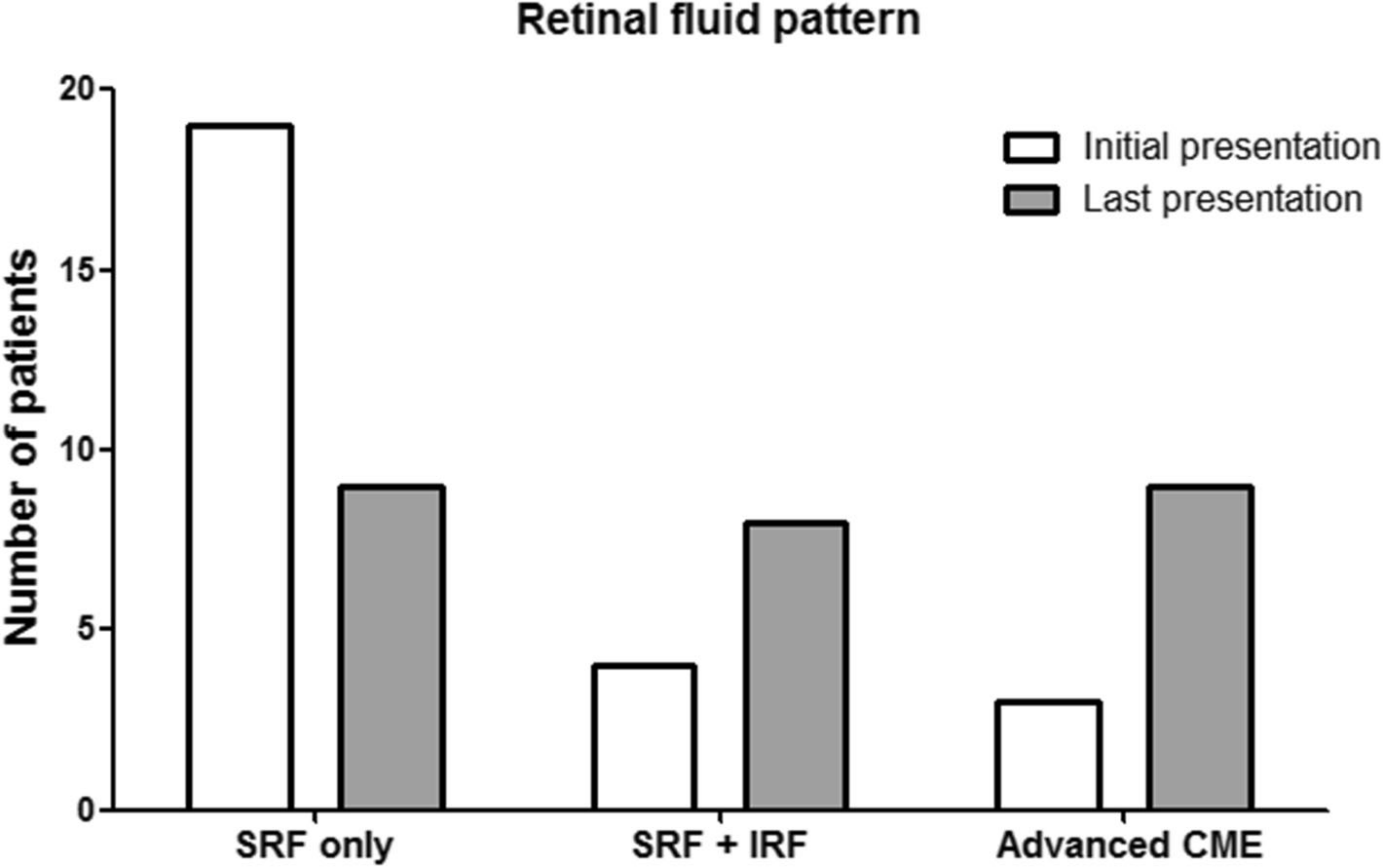
Changes in retinal fluid patterns over time in patients with circumscribed choroidal hemangioma. Among enrolled patients, patterns of retinal fluid at initial presentation were as follows: SRF-only in 19 patients (19/26, 73.1%), SRF and IRF combined in 4 patients (4/26, 15.4%), and advanced cystoid macular edema (CME) in 3 patients (3/26, 11.5%). Over time, changes in the pattern of retinal fluid occurred. The patterns of retinal fluid at last presentation were as follows: Advanced CME in 9 of 26 patients (9/26, 34.6%), SRF and IRF combined in 8 patients (8/26, 30.8%), and remained as SRF-only in 9 patients (9/26, 34.6%)

The pattern changes in retinal fluid associated with CCH are presented in Fig. 1. The pattern of retinal fluid at initial presentation was SRF-only in 19 patients (73.1%), SRF and IRF combined in 4 patients (15.4%), and advanced CME in 3 patients (11.5%).

Over time, the pattern of retinal fluid evolved. Among the patients with more than 48 months’ follow-up, the retinal fluid pattern progressed to an advanced CME pattern in 9 of 17 patients (52.9%), changed to SRF and IRF combined pattern in 6 patients (35.3%), and remained as SRF-only in only 2 patients (11.8%). IRF occurred in 2 out of 9 (22.2%) patients with 24 to 48 months’ follow-up.

Overall, the SRF-only pattern was observed mostly at initial presentation, whereas IRF occurred over time. The advanced CME pattern was observed mostly with patients who had long-term follow-up.

There was no association between the use of certain treatments and the development of advanced CME.

### Therapeutic effect on SRF or IRF according to treatment modalities

We examined the therapeutic response of retinal fluid according to each treatment modality performed as a primary therapy or secondary therapy.

Primary therapy was PDT in 9 patients (typical 7; enhanced 2), TTT in 7 patients and IVB in 10 patients. For PDT cases, ‘almost or complete’ response (mean score 1.0) was observed in 8 of 9 patients (typical 6; enhanced 2) (88.9%) and a ‘partial’ response (mean score 0.5) was observed in 1 patient (11.1%). For TTT cases, 3 patients (42.9%) showed ‘almost or complete’ resolution (mean score 1.0), 1 (14.3%) showed ‘partial’ resolution (mean score 0.5) and 3 (42.9%) showed ‘minimal or no’ effect (mean score 0.0). In IVB cases, 2 patients (20%) showed ‘partial to almost’ resolution (mean score 0.75), 3 patients showed ‘partial’ response (mean score 0.5) (30%) and 5 patients (50%) showed ‘minimal or no’ effect (mean score 0.0). PDT had a good therapeutic effect, and TTT and IVB showed modest therapeutic effects on retinal fluid with CCH.

Secondary therapy was performed as follows: PDT in 10 patients (typical 5; enhanced 5), TTT in 1 patient and IVB in 7 patients. TTT and IVB showed ‘minimal or no’ effect (mean score 0.0) in all patients. PDT showed ‘almost or complete’ resolution (mean score 1.0) in 5 patients (typical 2; enhanced 3) (50%), ‘partial to almost’ resolution (mean score 0.75) in 2 patients (typical 2) (20%) and ‘partial’ response (mean score 0.5) in 3 patients (typical 1; enhanced 2) (30%). Typical PDT and enhanced PDT showed similar effects. For all 3 treatment modalities, there was a lower therapeutic effect when they were applied as a secondary therapy compared to when they were used as a primary therapy.

Nine patients showed advanced CME during the follow-up period. IVBs were performed 19 times in 6 patients and showed ‘minimal or no’ effect (mean score 0.0) in all cases. TTT was performed once in 1 patient and showed ‘minimal or no’ effect (mean score 0.0). PDTs were performed 10 times (typical 6 times; enhanced 4 times) in 5 patients. Four out of 10 (40%) sessions showed ‘almost or complete’ response (mean score 1.0) and 2 out of 10 (20%) showed a ‘partial’ response (mean score 0.5), and 4 out of 10 (40%) showed ‘no or minimal’ effect (mean score 0.0). Enhanced PDT was not superior to typical PDT. IVB and TTT had no effect on advanced CME.

### Therapeutic effect on BCVA according to treatment modalities

Results from final BCVA compared with initial BCVA according to primary and secondary therapeutic modalities are presented in Table 2. Final BCVA improved in 9 out of 26 patients (34.6%), remained stable in 4 patients (15.4%), and deteriorated in 13 patients (50%) compared to initial BCVA. The mean final BCVA was lower than the initial BCVA with marginal significance [logMAR (Initial vs. Final): 0.79 ± 0.54 vs. 1.10 ± 0.91 (*p* = 0.064)].

**Table 2:** Final best corrected visual acuity (BCVA) compared with initial BCVA according to primary and secondary therapeutic modalities

	BCVA change			
Treatment modality	Improved	Stable	Worsen	Total number
P + A (A + P)	5 (3 + 2) (45.45%)	1 (0 + 1) (9.09%)	5 (3 + 2) (45.45%)	11
P	4 (1 + 3) (66.67%)	2 (1 + 1) (33.33%)		6
A		1 (1 + 0) (100%)		1
T + A (A + T)			6 (6 + 0) (100%)	6
T			2 (2 + 0) (100%)	2

Total number of patients (number of patients followed up over 48 months + number of patients followed up between 24 to 48 months)

*BCVA* best corrected visual acuity, *T* transpupillary thermotherapy, *P* photodynamic therapy, *A* intravitreal bevacizumab (Avastin) injection

We further examined BCVA changes according to each treatment modality. Among 17 patients followed up over 48 months, as primary and secondary therapies, 8 patients received PDT at least once and 8 patients received TTT at least once. There was no significant difference in age, tumour position, and tumour size between the PDT group and the TTT group. Comparing final BCVA with initial BCVA, in PDT cases, 4 out of 8 patients (50%) showed an improvement in BCVA, 1 patient (12.5%) remained stable and 3 patients (37.5%) deteriorated. Whereas, all patients that received TTT showed deterioration in their BCVA (8/8 = 100%).

All 9 patients who were followed up for 24 to 48 months received PDT plus IVB (or IVB plus PDT). Comparing final BCVA with initial BCVA, 5 out of 9 patients (55.6%) showed improvement in their BCVA, 2 patients (22.2%) remained stable, and 2 patients’ (22.2%) BCVA deteriorated. The mean final BCVA was improved when compared to the initial BCVA, however, this was not statistically significant (*p* = 0.473).

### Cases

**Fig. 2:**
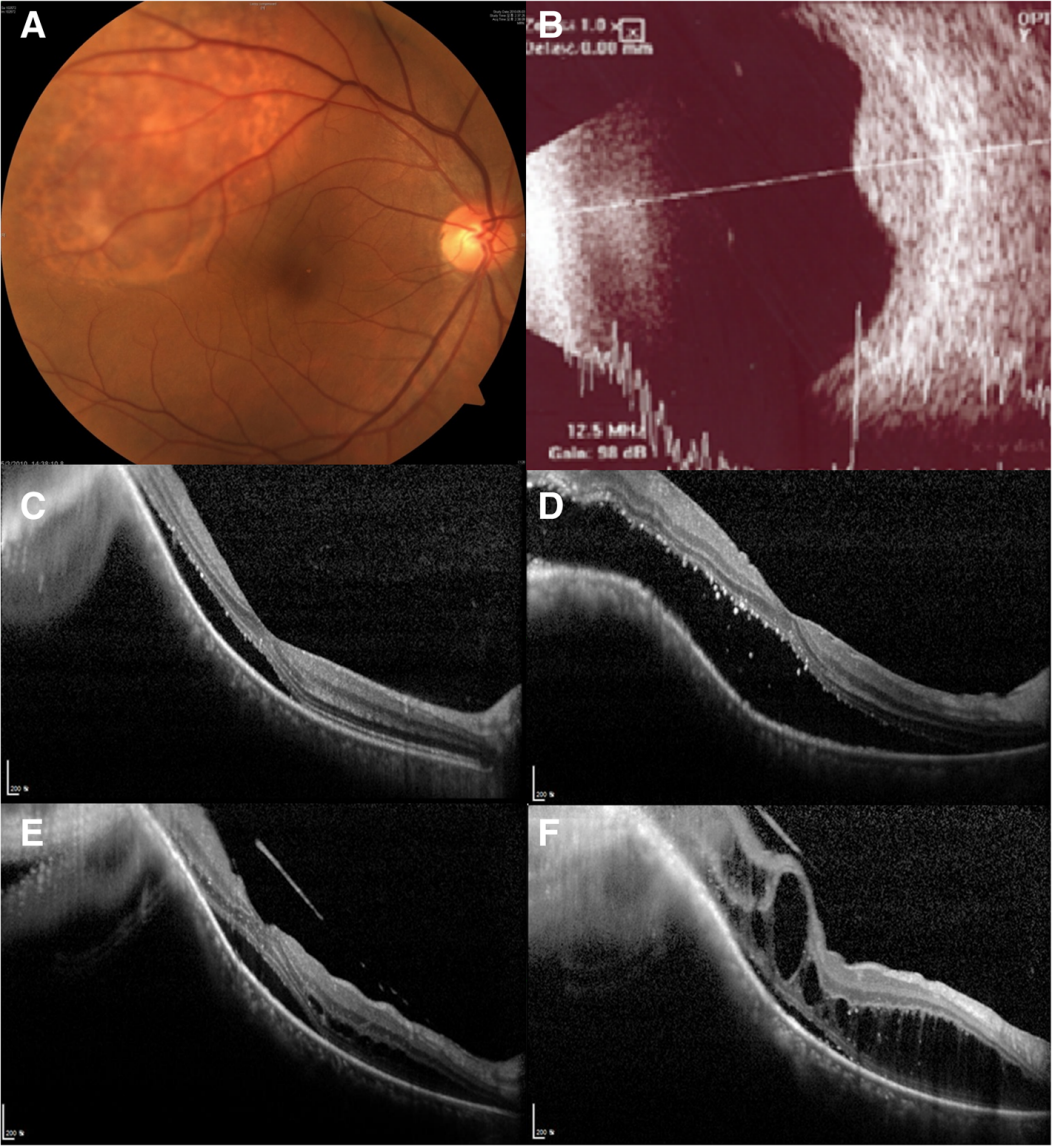
A representative case of circumscribed choroidal hemangioma showing the change in retinal fluid over time. A 48-year-old woman (Case 7) presented with blurred vision of the right eye. Her initial best corrected visual acuity (BCVA) was 0.63. (**a**) On fundus photography, an orange and red-colored subretinal mass was observed supero-temporally to the macula. (**b**) B-scan ultrasonography showed a hyperreflective mass with a height of 4.77 mm and base diameter of 11.07 mm. (**c**) Shallow subretinal fluid involving the fovea was observed along with elevation of the choroid by a mass lesion on optical coherence tomography. (**d**) During the first 6 months, despite a single intravitreal bevacizumab (IVB) injection, the subretinal fluid (SRF) increased. Thereafter, a second IVB injection and single session of transpupillary thermotherapy (TTT) were performed; however, there was no significant improvement in therapeutic effect. Subsequently, a single session of photodynamic therapy (PDT) and enhanced PDT were performed and the SRF completely resolved. However, the subsequent BCVA was 0.04. (**e**) After 2 years of follow-up, SRF and intraretinal fluid then developed. (**f**) Six months later, an advanced cystoid macular edema pattern was observed

**Fig. 3:**
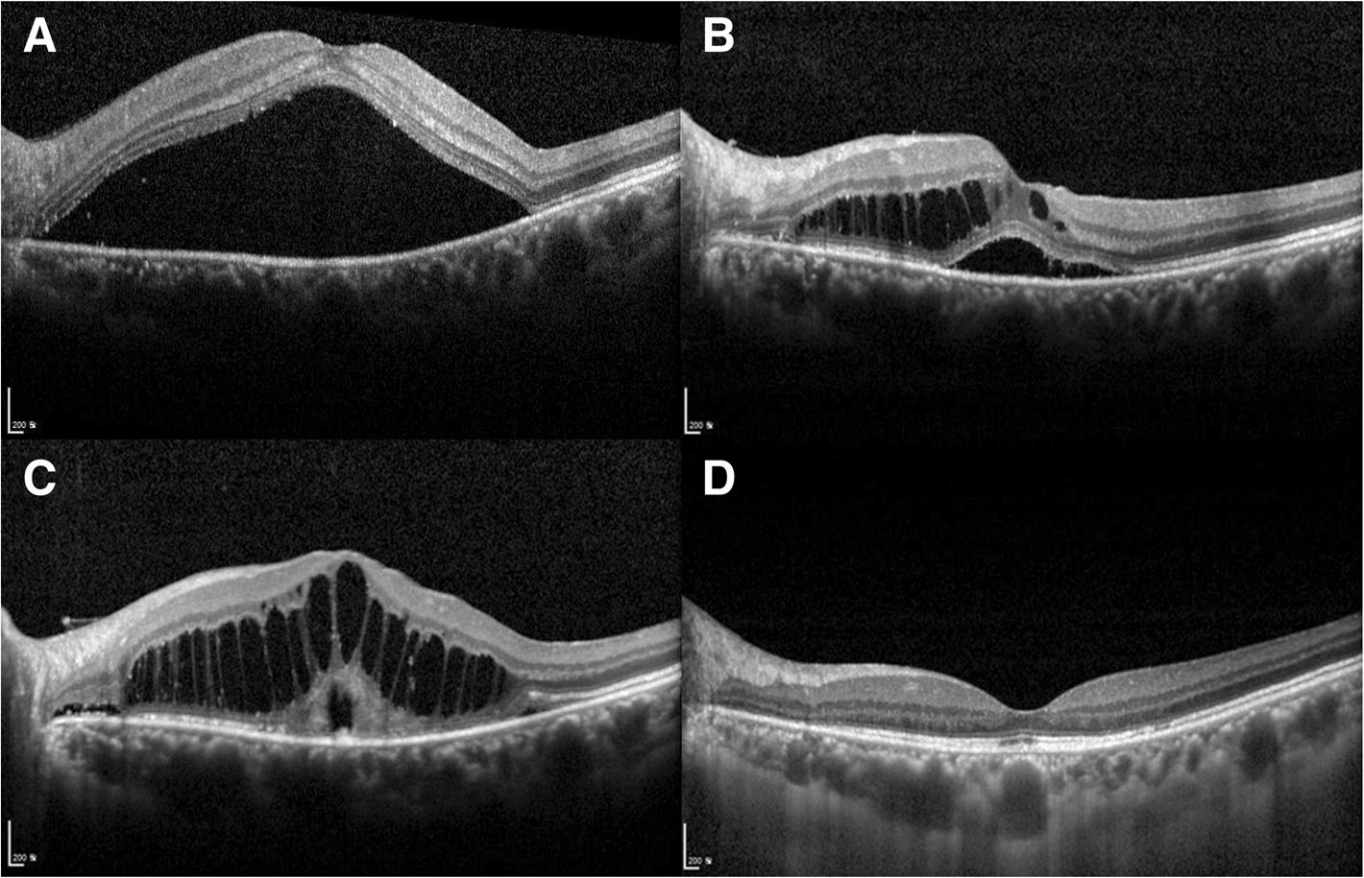
A representative case of circumscribed choroidal hemangioma where complete resolution of advanced cystoid macular edema (CME) occurred using photodynamic therapy (PDT). A 44-year-old woman (Case 6) presented with blurred vision of the left eye. Her initial best corrected visual acuity (BCVA) was 0.63. At initial presentation, minimal subretinal fluid (SRF) was present. (**a**) After 8 months, the BCVA decreased to 0.025, and a large serous retinal detachment (SRD) developed. A single intravitreal bevacizumab injection was performed, and the SRD completely resolved. (**b**) After 28 months, SRF and intraretinal fluid (IRF) occurred. A second intravitreal bevacizumab injection was performed; however, the fluid only partially resolved. (**c**) Three months later, advanced CME developed. Two subsequent PDTs were performed, and complete resolution occurred. Thereafter, there was a minor recurrence of SRF and IRF, and enhanced PDT was performed, and the fluid was again completely resolved. (**d**) After more than 3 years of follow-up, the fluid did not recur

We present two representative cases demonstrating the pattern of change in retinal fluid associated with CCH. In both cases, SRF-only patterns were noted at initial presentation and over time, SRF transitioned to IRF, eventually progressing to advanced CME, despite various treatment efforts. In one case (Fig. 2; Case 7), the retinal fluid responded well to PDT initially, however, the fluid eventually returned and subsequently progressed to the advanced CME pattern. In the other case (Fig. 3; Case 6), the retinal fluid was completely resolved following multiple treatments with PDT, and there was no recurrence during the more than 3 years of follow-up.

## Discussion

We investigated the changes in retinal fluid patterns and the therapeutic response to various treatment modalities in patients with symptomatic CCH who underwent long-term follow-up. There have been no studies that focus on changes in retinal fluid patterns associated with CCH.

The SRF-pattern was mainly observed in the early stage, followed by SRF and IRF combined, and eventually, an advanced CME pattern was established over time. During the follow-up period, advanced CME occurred regardless of the treatment modality. Despite treatment efforts, the occurrence of CME could not be prevented. Patients with advanced CME at initial presentation may be assumed to have had long-lasting retinal fluid along with CCH.

The pathophysiology of CME in CCH is poorly understood. CME often occurs due to breakdown of the inner blood–retina barrier and is not an uncommon manifestation of diabetic retinopathy, retinal vein occlusion, and inflammatory diseases of the posterior segment. Less commonly, CME is the result of incompetence of the outer blood–retina barrier [20]. In long-standing central serous chorioretinopathy, some eyes will develop CME [21, 22]. In the case of CME following a long lasting SRF, it may be related to alteration of the external limiting membrane (ELM), which is the linear aggregate of junctions between the outer portions of Müller cells and the inner segments of the photoreceptors. The ELM may serve as a barrier for fluid leaving the retina to be pumped from the subretinal space by the retinal pigment epithelium. When the ELM is intact, fluid from below the retina can cause serous detachment of the retina, and when the ELM is defective, there may be passage of fluid into the outer retina [23].

Among the three treatment modalities reported in this study, PDT showed the greatest treatment efficacy, and TTT and IVB showed moderate efficacy for retinal fluid reduction as a primary therapy. As secondary therapies, the efficacy of all 3 treatment modalities was reduced. As the retinal fluid with the tumour became older or recurred, it appears to have become refractory to various treatments. Early and appropriate treatment of CCH is important.

Several years ago, our group reported the efficacy of IVB in the treatment of 12 symptomatic CCH patients [8]. IVB led to the resolution of serous macular detachment and improvement of visual acuity in 5 of 9 patients. However, its duration of treatment effectiveness seemed to be relatively short. Similar results were observed in this study. Although IVB may have the advantages of greater feasibility and lower cost than PDT or TTT, IVB monotherapy seems to be insufficiently effective to manage CCH completely. Repeated IVB therapy should not delay PDT therapy.

Our study findings may have clinical significance because different therapeutic responses were observed depending on the retinal fluid pattern. CME has been considered a poor prognostic factor in CCH [2, 24]. A previous study by our group showed that CME does not respond effectively to IVB [8]. In this study, after formation of advanced CME, IVB was not effective in all 19 applications, and PDT was effective in half of the cases. The Shields group also previously reported a case where PDT was effective in CME with CCH [24].

It is not clear whether the treatment response to advanced CME is poor due to inherent characteristics of the CME or as a result of long-standing CCH. Alternatively, the poor response may be due to the long-term presence of retinal fluid or damage to the structure of the retinal by previous treatments. Each of these reasons alone or in combination may explain this observation.

According to recent research in CCH treatment, PDT has emerged as the treatment of choice with high rates of tumour regression, fluid resorption, and minimal complications [4–7, 18]. Our findings agree with this research as PDT showed the most promising results not only as a primary therapy, but also after the formation of advanced CME.

## Conclusion

Among patients with long-term follow-up, over half of the patients treated with PDT showed improvement in their BCVAs. In conclusion, PDT appears to be an effective treatment for symptomatic retinal fluid associated with CCH.

## Abbreviations

BCVA: Best corrected visual acuities
CCH: circumscribed choroidal hemangioma
CME: cystoid macular edema
DD: disc diameter
ELM: external limiting membrane
ICGA: indocyanine green angiography
IRF: intraretinal fluid
IVB: intravitreal bevacizumab injection
LBD: largest base diameter
LogMAR: log of the minimum angle of resolution
OCT: optical coherence tomography
PDT: photodynamic therapy
SMD: serous macular detachment
SRF: subretinal fluid
TTT: transpupillary thermotherapy
VEGF: vascular endothelial growth factor
